# Transcription Factor Spo0A Regulates the Biosynthesis of Difficidin in Bacillus amyloliquefaciens

**DOI:** 10.1128/spectrum.01044-23

**Published:** 2023-07-11

**Authors:** Na Liu, Huiwan Sun, Zhengyu Tang, Yuqing Zheng, Gaofu Qi, Xiuyun Zhao

**Affiliations:** a College of Life Science and Technology, Huazhong Agricultural University, Wuhan, Hubei Province, China; University of Minnesota Twin Cities

**Keywords:** *Bacillus amyloliquefaciens*, *Ralstonia solanacearum*, difficidin, Spo0A, bacillibactin

## Abstract

Bacillus amyloliquefaciens WH1 produces multiple antibiotics with antimicrobial activity and can control bacterial wilt disease caused by Ralstonia solanacearum. Antibacterial substances produced by WH1 and the regulation mechanism are unknown. In this study, it was found that difficidin, and to a minor extent bacillibactin, exhibited antibacterial activity against R. solanacearum. Lipopeptides, macrolactin, bacillaene, and bacilysin had no antibacterial activity. Ferric iron uptake transcriptional regulator Fur bound the promoter region of the *dhb* gene cluster of bacillibactin biosynthesis. Mutant Δ*fur* showed a higher bacillibactin production and its antibacterial activity increased by 27% than wild-type WH1. Difficidin inhibited R. solanacearum growth and disrupted the integrity of the cells. Lack of transcription factor Spo0A abolished difficidin biosynthesis. Spo0A bound the promoter region of the *dfn* gene cluster of difficidin biosynthesis. Changing phosphorylation levels of Spo0A via deletion of phosphatase gene *spo0E* and histidine kinases genes *kinA* and *kinD* affected the biosynthesis of difficidin. Deletion of *spo0E* increased the phosphorylation level of Spo0A and consequently improved the difficidin production. The antibacterial activity of mutant Δ*spo0E* and Δ*kinA* increased by 12% and 19%. The antibacterial activity of mutant Δ*kinD* decreased by 28%. Collectively, WH1 produced difficidin to disrupt the cell of R. solanacearum and secreted siderophore bacillibactin to compete for ferric iron. Spo0A regulated difficidin biosynthesis. Spo0A regulates quorum-sensing responses and controls the biosynthesis of secondary metabolites in *B. amyloliquefaciens*. This study has important findings in the regulation mechanism of antibiotic synthesis and helps to improve antibiotic yield in *Bacillus*.

**IMPORTANCE** Pathogen R. solanacearum causes bacterial wilt disease in many crops. There is no chemical bactericide that can control bacterial wilt disease. It is vital to find antagonistic microorganisms and antibacterial substances that can efficiently control bacterial wilt disease. *B. amyloliquefaciens* WH1 could inhibit the growth of R. solanacearum. Via genetic mutation, it was found that difficidin and to a minor extent bacillibactin produced by WH1 acted efficiently against R. solanacearum. The transcription factor Spo0A regulated the synthesis of difficidin. Phosphorylation of Spo0A affected the production of difficidin. Increasing the phosphorylation level of Spo0A improved the difficidin production and antibacterial activity. In-depth analysis of the regulation mechanism of antibiotic difficidin is meaningful for enhancing the control efficiency of WH1. *B. amyloliquefaciens* WH1 and the antibacterial substances have vast application potential in controlling bacterial wilt disease.

## INTRODUCTION

The devastating bacterial wilt disease is caused by Ralstonia solanacearum, which infects a broad range of crops in tropical and subtropical regions (1, 2). Bacterial wilt disease seriously threatens plant growth and consequently leads to great economic losses worldwide. R. solanacearum constantly infects Solanaceae crops (e.g., potato, eggplant, tobacco, tomato, banana) in southern China and causes increasing economic loss over time (3, 4). For controlling bacterial wilt, traditional agricultural practices, such as chemical bactericide application, field sanitation, and crop rotation, have been widely applied. Bacterial wilt disease is difficult to control due to the high variability of the pathogen races and the appearance of bactericide-resistant pathogens (5, 6). Currently, many researchers have focused on developing microbial agents for control of bacterial wilt because the application of biocontrol agents has been verified as an efficient and environment-friendly method in recent years (2).

*Bacillus* species have been used as effective biocontrol agents to reduce damages caused by bacterial wilt (6) because they can produce a wide range of antibiotics and form endospores to survive under adverse environmental conditions. Many *Bacillus* species possess antibacterial activity, including *B. amyloliquefaciens*, Bacillus subtilis, Bacillus vallismortis, and Bacillus velezensis (2). The biocontrol effects of *Bacillus* agents are primarily associated with the production of various bioactive molecules (6). Lipopeptides are one type of antimicrobial substance isolated from *Bacillus*. For example, *B. velezensis* and *B. amyloliquefaciens* can produce lipopeptides such as surfactin, iturin, and fengycin against bacterial wilt (1, 6, 7). Fengycin is active against R. solanacearum and effectively controls tomato bacterial wilt with a biocontrol efficiency of 97.6% (5, 6). Iturin exhibits weak antibacterial activity, and surfactin displays effective bactericidal activity (6). *Bacillus* species also produce bioactive peptides and polyketide antibiotics such as bacillaene, difficidin, and macrolactin (2, 3, 8–10). Difficidin is a polyketide class of polyene, which is biosynthesized by type I polyketide synthase (PKS) encoded by the *dfn* gene cluster (11, 12). Synthesis of lipopeptides and polyketides is dependent on 4′-phosphopantetheinyl transferase that is encoded by gene *sfp* (11). Dipeptides and iron siderophores (e.g., bacillibactin) are low-molecular compounds produced by *Bacillus* species (3). Small molecule iron-chelators siderophores produced by rhizobacteria play an important role in inhibiting pathogens.

Surfactin produced by *Bacillus* triggers quorum-sensing responses such as biofilm formation and sporulation (13). First, surfactin activates the histidine kinases (KinA-E), which are autophosphorylated. Second, the phosphorylated kinases give the phosphate group to regulator Spo0A via the phosphate transfer proteins Spo0F and Spo0B. Finally, Spo0A~P directly or indirectly controls the transcription of more than 500 genes in *Bacillus*, such as the genes involved in biofilm formation, sporulation and secondary metabolites synthesis (13). Conversely, Spo0A~P can be dephosphorylated by phosphatases such as RapA and Spo0E, etc. (14). Many studies have revealed that Spo0A regulates the production of industrial goods in *Bacillus*. The transcription factor Spo0A regulates the biosynthesis of many secondary metabolites. For example, the deletion of *spo0A* causes low or null production of bacitracin (15–17). The deletion of *spo0A* has resulted in a significant decrease in iturin and fengycin production in *B. amyloliquefacien* WH1 (18).

*B. amyloliquefaciens* exhibits antagonistic activity against a broad spectrum of pathogens (19). The gene clusters involved in the biosynthesis of lipopeptides, macrolactin, bacillaene, bacilysin, difficidin, and bacillibactin were simultaneously identified in the genome of *B. amyloliquefaciens* WH1. WH1 has excellent antibacterial and antifungal activities (18). In this study, we try to identify the antibacterial compounds produced by WH1 and investigate the regulation mechanism controlling antibiotics synthesis.

## RESULTS

### Lipopeptides, bacilysin, bacillaene, and macrolactin have no antibacterial activity.

**FIG 1:**
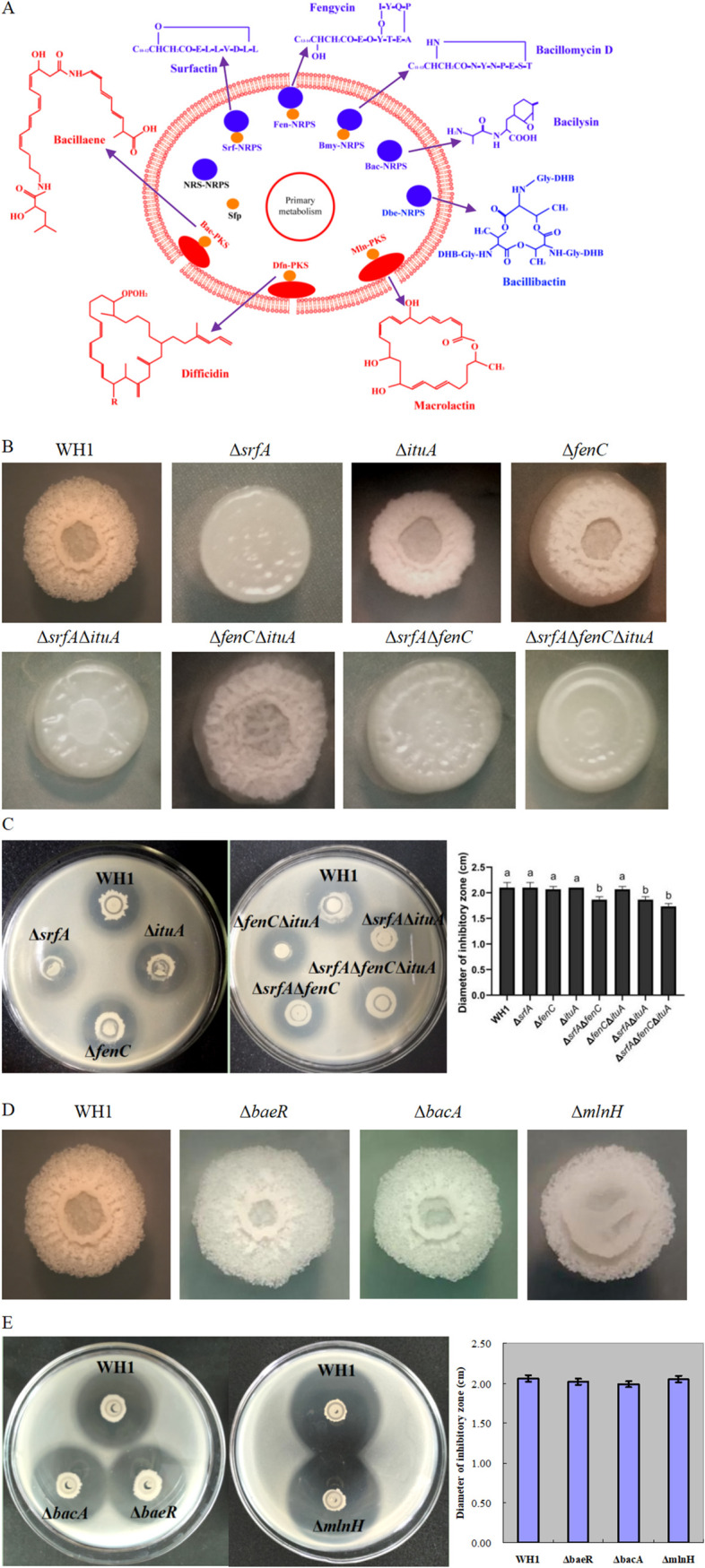
Antibacterial activity of mutants deficient in biosynthesis of lipopeptides, bacilysin, bacillaene, and macrolactin is similar to wild-type WH1. (A) Schematic diagram of antimicrobial secondary metabolites (NPRS and PKS, synthetic pathway) produced by *B. amyloliquefaciens* WH1. (B) Colony morphology of mutants deleting the gene cluster of lipopeptides biosynthesis. (C) Antibacterial activity of mutants deleting the gene cluster of lipopeptides biosynthesis. (D) Colony morphology of mutants Δ*bacA*, Δ*baeR*, and Δ*mlnH*. (E) Antibacterial activity of mutants Δ*bacA*, Δ*baeR*, and Δ*mlnH* toward R. solanacearum. Different letters on bars indicate significant differences among groups. The same image is being utilized to represent the colony of the wild-type WH1 in Fig. 1B, 1D, 2A, 2B, 2C, 3A, and 3C because they are from the same internally controlled experiment.

The biosynthesis gene clusters of lipopeptides (i.e., surfactin, iturin, fengycin) were identified in the WH1 genome (Fig. 1). The antibacterial activity of the mutants deficient in the production of surfactin (Δ*srfA*), iturin (Δ*ituA*), and fengycin (Δ*fenC*) was determined, respectively. Mutants Δ*srfA* (diameter of inhibition zone [DIZ] = 2.07 cm), Δ*ituA* (DIZ = 2.14 cm), and Δ*fenC* (DIZ = 2.04 cm) showed similar antibacterial activity to wild-type WH1 (DIZ = 2.11 cm), indicating that blocking the biosynthesis of single lipopeptide did not influence the antibacterial activity. The antibacterial activity of double-mutation strains Δ*srfA*Δ*ituA* (DIZ = 1.8 cm) and Δ*srfA*Δ*fenC* (DIZ = 1.81 cm) decreased by 15% and 14%, respectively. Moreover, the antibacterial activity of mutant Δ*ituA*Δ*fenC* (DIZ = 2.01 cm) was similar to wild-type WH1. The antibacterial activity of triple-mutation strain Δ*srfA*Δ*ituA*Δ*fenC* deficient in biosynthesis of three lipopeptides decreased by 18% (DIZ = 1.73 cm). Surfactin had no direct activity against R. solanacearum but was necessary for cell growth. Therefore, the mutants Δ*srfA*Δ*ituA*, Δ*srfA*Δ*fenC*, and Δ*srfA*Δ*fenC*Δ*ituA* had a weaker growth than wild-type WH1, and as a result showed a weaker antibacterial activity (20). The wild-type WH1 colony formed wrinkles and bulges. Meanwhile, the colonies of mutants deleting *srfA* (Δ*srfA*, Δ*srfA*Δ*ituA*, Δ*srfA*Δ*fenC*, Δ*srfA*Δ*fenC*Δ*ituA*) were smooth, indicating a lack of surfactin hindered biofilm formation.

The biosynthesis gene clusters of bacillaene (*baeR*), bacilysin (*bacA*), and macrolactin (*mlnH*) were genetically mutated, respectively (Fig. 1). The mutants Δ*baeR* (DIZ = 2.04 cm), Δ*bacA* (DIZ = 2.03 cm), and Δ*mlnH* (DIZ = 2.08 cm) showed similar antibacterial activity to wild-type WH1 (DIZ = 2.08 cm), indicating that bacillaen, bacilysin, and macrolactin had no antibacterial activity toward R. solanacearum.

### Difficidin is mainly responsible for the antibacterial activity toward R. solanacearum.

**FIG 2:**
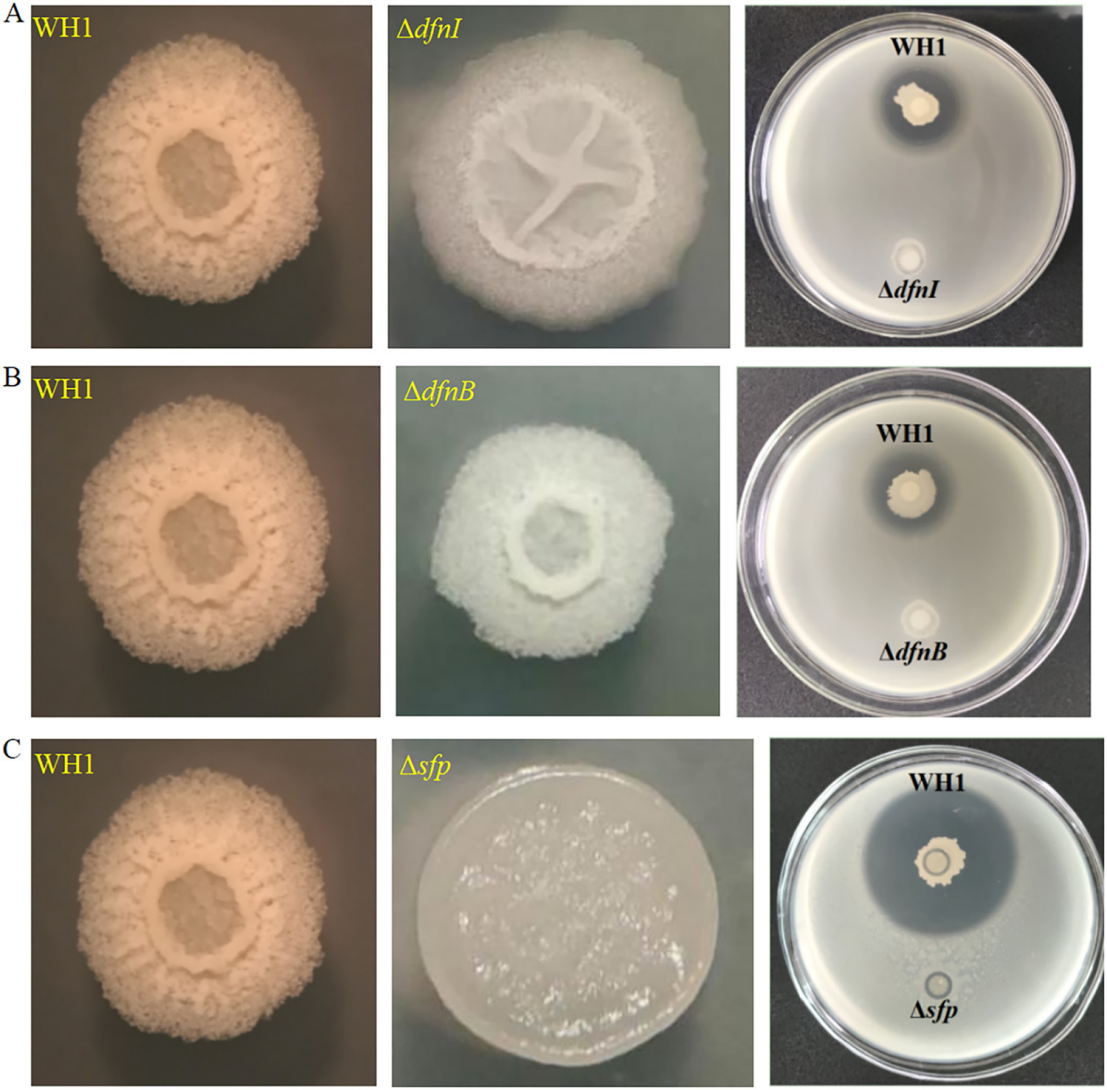
Antibacterial activity of mutants with a deficiency in difficidin biosynthesis disappears. Colony morphology and antibacterial activity of mutants Δ*dfnI* (A), Δ*dfnB* (B), and Δ*sfp* (C). The same image is being utilized to represent the colony of the wild-type WH1 in Fig. 1B, 1D, 2A, 2B, 2C, 3A, and 3C because they are from the same internally controlled experiment.

The gene *dfnI* involved in difficidin biosynthesis was genetically mutated. The antibacterial activity of mutant Δ*dfnI* disappeared (DIZ = 0 cm) (Fig. 2), indicating the antibacterial activity of WH1 toward R. solanacearum was mainly attributed to difficidin. The *dfnB* gene belonged to the *dfn* gene cluster of difficidin biosynthesis. The mutant Δ*dfnB* (DIZ = 0 cm) lost the antagonistic activity like Δ*dfnI*, further confirming that the main antibacterial substance produced by WH1 was difficidin. The gene *sfp* encodes 4’-phosphopantetheine transferase, which is essential for the biosynthesis of difficidin. The antibacterial activity of mutant Δ*sfp* decreased by 91% (DIZ = 0.2 cm). The colony of Δ*sfp* was smooth, indicating biofilm formation was suppressed in mutant cells.

### Bacillibactin exhibits slight antibacterial activity.

**FIG 3:**
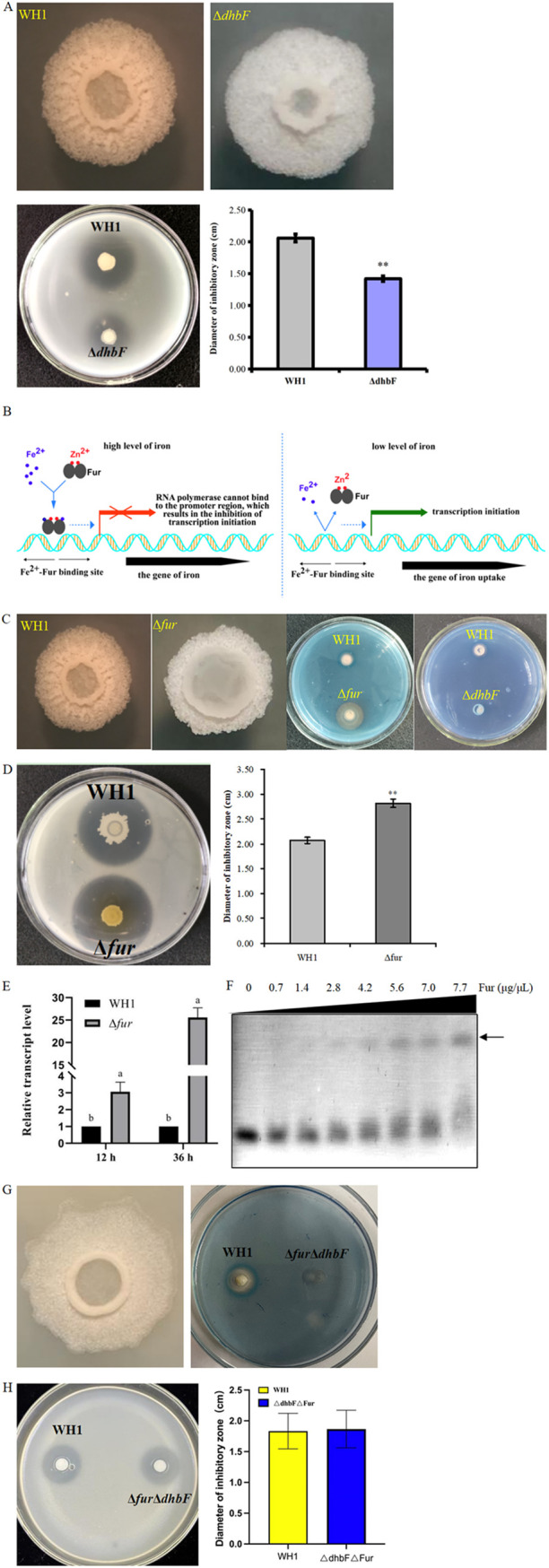
Bacillibactin exhibits antibacterial activity against R. solanacearum. (A) Colony morphology and antibacterial activity of mutant Δ*dhbF*. (B) Schematic diagram showing how Fur regulates the biosynthesis of bacillibactin. In the presence of sufficient iron, the regulator Fur binds Fe^2+^ and Zn^2+^, thus suppressing the transcription of the *dhb* gene cluster, and bacillibactin biosynthesis is blocked; When the iron is deficient in the environment, Fe^2+^ is dissociated from Fur, transcription of the *dhb* gene cluster is activated, and bacillibactin is synthesized. (C) Colony morphology and production of bacillibactin in mutant Δ*fur*. (D) Comparing antibacterial activity of mutant Δ*fur* and wild-type WH1. (E) Comparing the transcription level of gene *dhbF* in mutant Δ*fur* and wild-type WH1. (F) EMSA showing protein Fur binds promoter P*_dhb_*. (G) Colony morphology and bacillibactin production in mutant Δ*fur*Δ*dhbF*. (H) Comparing antibacterial activity of mutant Δ*fur*Δ*dhbF* and wild-type WH1. Different letters on bars indicate significant differences among groups. The same image is being utilized to represent the colony of the wild-type WH1 in Fig. 1B, 1D, 2A, 2B, 2C, 3A, and 3C because they are from the same internally controlled experiment.

The gene *dhbF* involved in bacillibactin biosynthesis was genetically mutated. The antibacterial activity of mutant Δ*dhbF* (DIZ = 1.42 cm) decreased by 31% than wild-type WH1 (DIZ = 2.06 cm) (Fig. 3). Therefore, bacillibactin exhibited certain antibacterial activity toward R. solanacearum. Bacillibactin partly contributed to the antibacterial activity of WH1 considering most of the antibacterial activity of Δ*dhbF* was still preserved.

Previous studies have shown that the regulator Fur suppresses the transcription of the *dhb* gene cluster of bacillibactin biosynthesis (21). The antibacterial activity of mutant Δ*fur* (DIZ = 2.82 cm) increased by 27% than wild-type WH1 (DIZ = 2.07 cm). The transcriptional level of *dhbF* in Δ*fur* increased by 20 times at the stationary phase (cell growing 36 h) compared to that in the wild-type WH1. The antibacterial activity of Δ*fur*Δ*dhbF* (DIZ = 1.83 cm) was similar to wild-type WH1 (DIZ = 1.82 cm). This result indicated that the increased antibacterial activity of Δ*fur* was attributed to the increase in bacillibactin production. The recombinant protein Fur was expressed in Escherichia coli and purified by Ni-NAT column (Fig. S1). EMSA revealed that the Fur protein was binding to the promoter region of the *dhb* gene cluster.

### Difficidin shows strong antibacterial activity and kills R. solanacearum.

**FIG 4:**
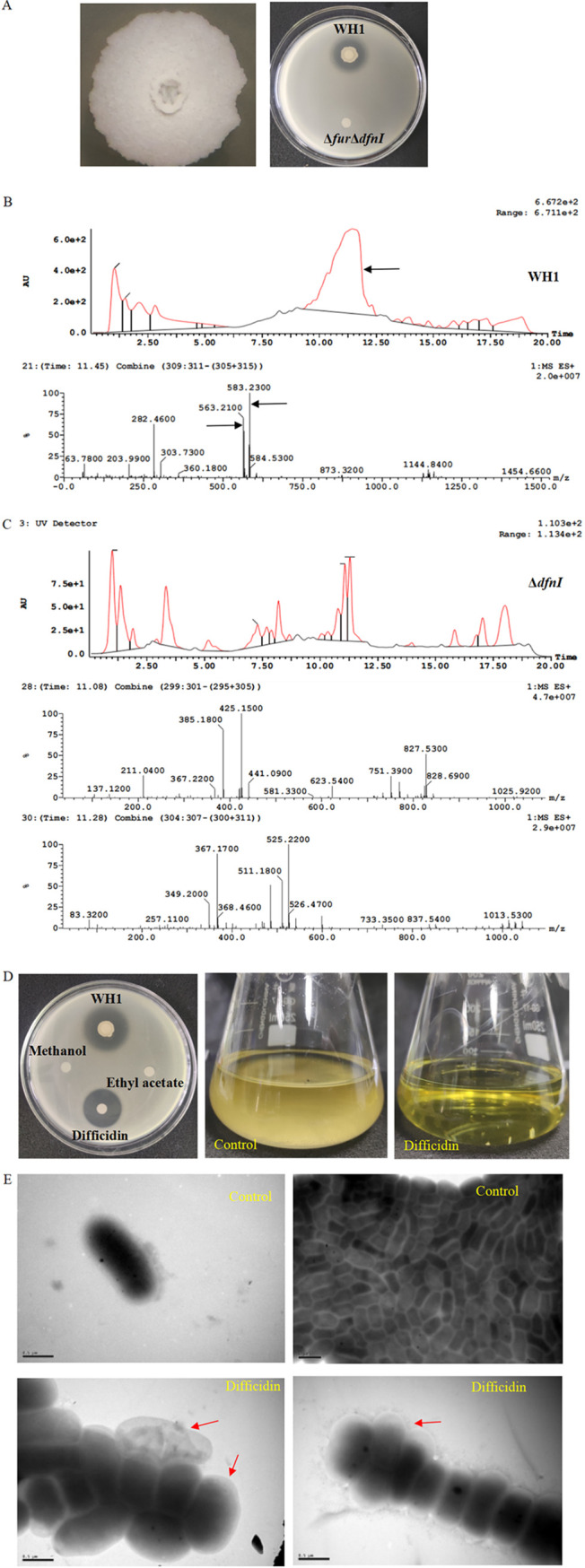
Difficidin inhibits R. solanacearum growth and disrupts cells of the pathogen. (A) Colony morphology and antibacterial activity of mutant Δ*fur*Δ*dfnI*. (B) Purification and characterization of difficidin from WH1 broth by HPLC and mass spectrometry. Arrow directs the peak containing difficidin. The *m/z* values of 583.23 and 563.21 are characterized as difficidin bound with single and double K^+^ via phosphate group, respectively. (C) Difficidin is undetectable in the broth of mutant Δ*dfnI*. (D) Detecting antibacterial activity of difficidin. Left: difficidin inhibits the growth of R. solanacearum on plate; Right: difficidin inhibits the growth of R. solanacearum in broth. Bacterial culture is untreated with difficidin is used as the control. (E) Cell morphology of R. solanacearum treated with difficidin. Arrows direct the changes in the cell morphology. Cell untreated with difficidin was used as the control.

The antibacterial activity of mutant Δ*fur*Δ*dfnI* (DIZ = 0 cm) dramatically disappeared (Fig. 4), similar to the antibacterial activity of Δ*dfnI* but very different from Δ*fur*, further clarifying the main antibacterial compound was difficidin rather than bacillibactin.

The difficidin was extracted from wild-type WH1 broth, showing a peak at 11.45 min after separation by high-performance liquid chromatography (HPLC). This peak disappeared in the extracts of mutant Δ*dfnI*. Based on mass spectrometry analysis, the compounds with *m/z* values of 583.23 and 563.21 were characterized as difficidin bound with single and double K^+^ via phosphate group, respectively.

R. solanacearum growth was completely inhibited by difficidin. R. solanacearum cells were swelled, intracellular cytoplasm leaked out, cells ultimately died, and the residual cell wall made the cell like a ghost (directed by red arrows).

### Transcription factor Spo0A is essential for the biosynthesis of difficidin.

**FIG 5:**
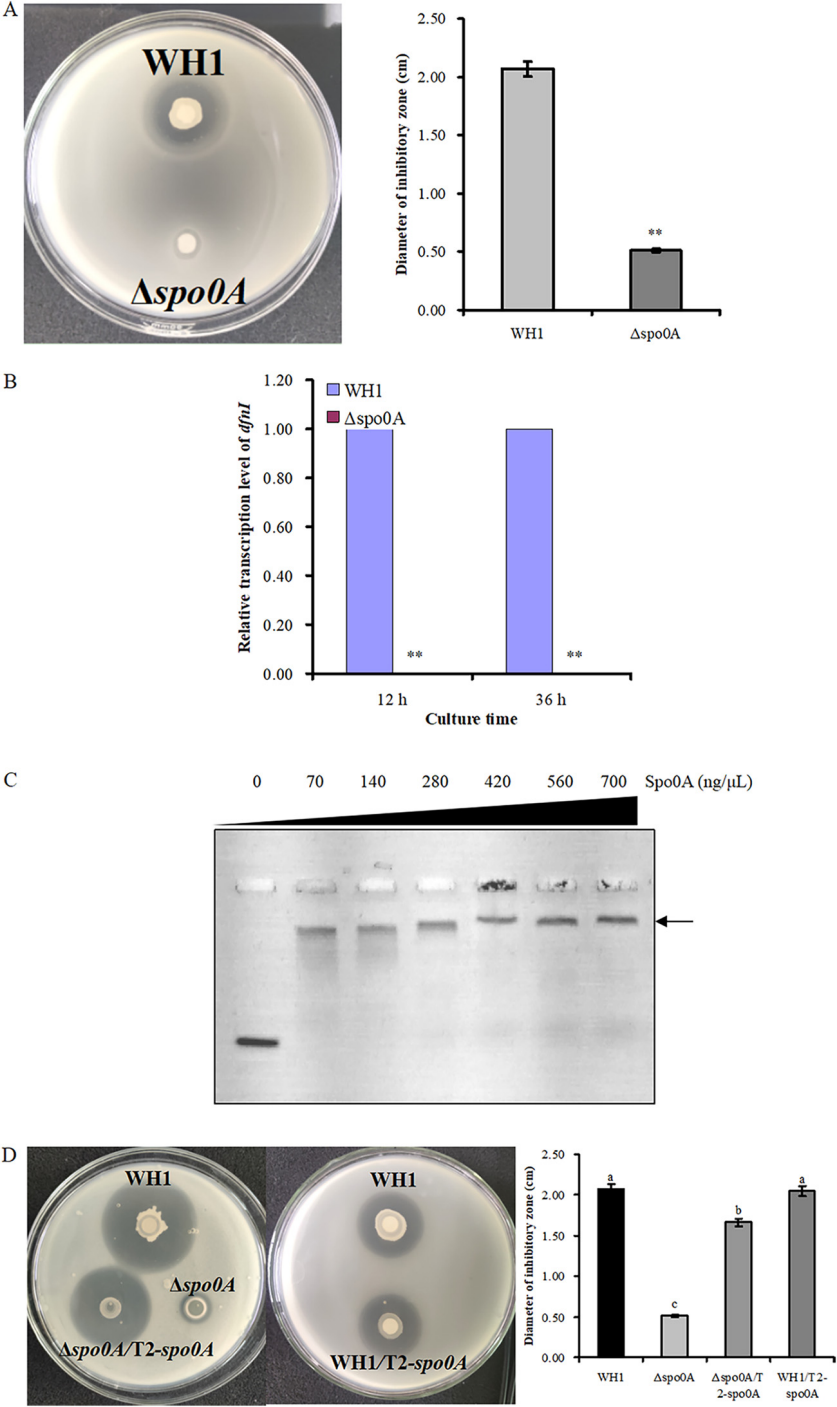
Spo0A is essential for difficidin biosynthesis. (A) Comparing antibacterial activity of mutant Δ*spo0A* and wild-type WH1. (B) Detecting the transcription level of gene *dfnI* by qRT-PCR. (C) EMSA showing interactions between Spo0A and promoter P*_dfn_*. Arrow directs the shift of bands. (D) Comparing the antibacterial activity of wild-type WH1, Δ*spo0A*, Δ*spo0A*/T2-*spo0A* and WH1/T2-*spo0A*. Different letters on bars indicate significant differences among groups.

The antibacterial activity of mutant Δ*spo0A* decreased by 75% (DIZ = 0.51 cm) (Fig. 5), indicating Spo0A was essential for the biosynthesis of antibacterial substances. The antibacterial activity of the complementary strain Δ*spo0A*/T2-Δ*spo0A* (DIZ = 1.66 cm) was restored although it was still weaker than the wild-type WH1 (DIZ = 2.07 cm). However, the antibacterial activity of overexpression strain WH1/T2-*spo0A* (DIZ = 2.05 cm) did not increase compared to the wild-type WH1. The results confirmed that Spo0A was essential for the biosynthesis of antibacterial substances. The gene *dfnI* belonged to the *dfn* gene cluster of difficidin biosynthesis. The transcription of *dfnI* was undetectable in Δ*spo0A* both at the exponential phase (12 h) and stationary phase (36 h), further confirming that Spo0A was essential for the biosynthesis of difficidin.

The recombinant protein Spo0A was expressed in E. coli and purified by the Ni-NAT column (Fig. S1). EMSA revealed that Spo0A was binding to the *dfn* promoter. Accompanying by increasing Spo0A concentration, more probes bound with Spo0A showed an obvious shift in the gel. The result inferred that Spo0A regulated the transcription of the *dfn* gene cluster.

### Tuning phosphorylation levels of Spo0A affects difficidin production.

**FIG 6:**
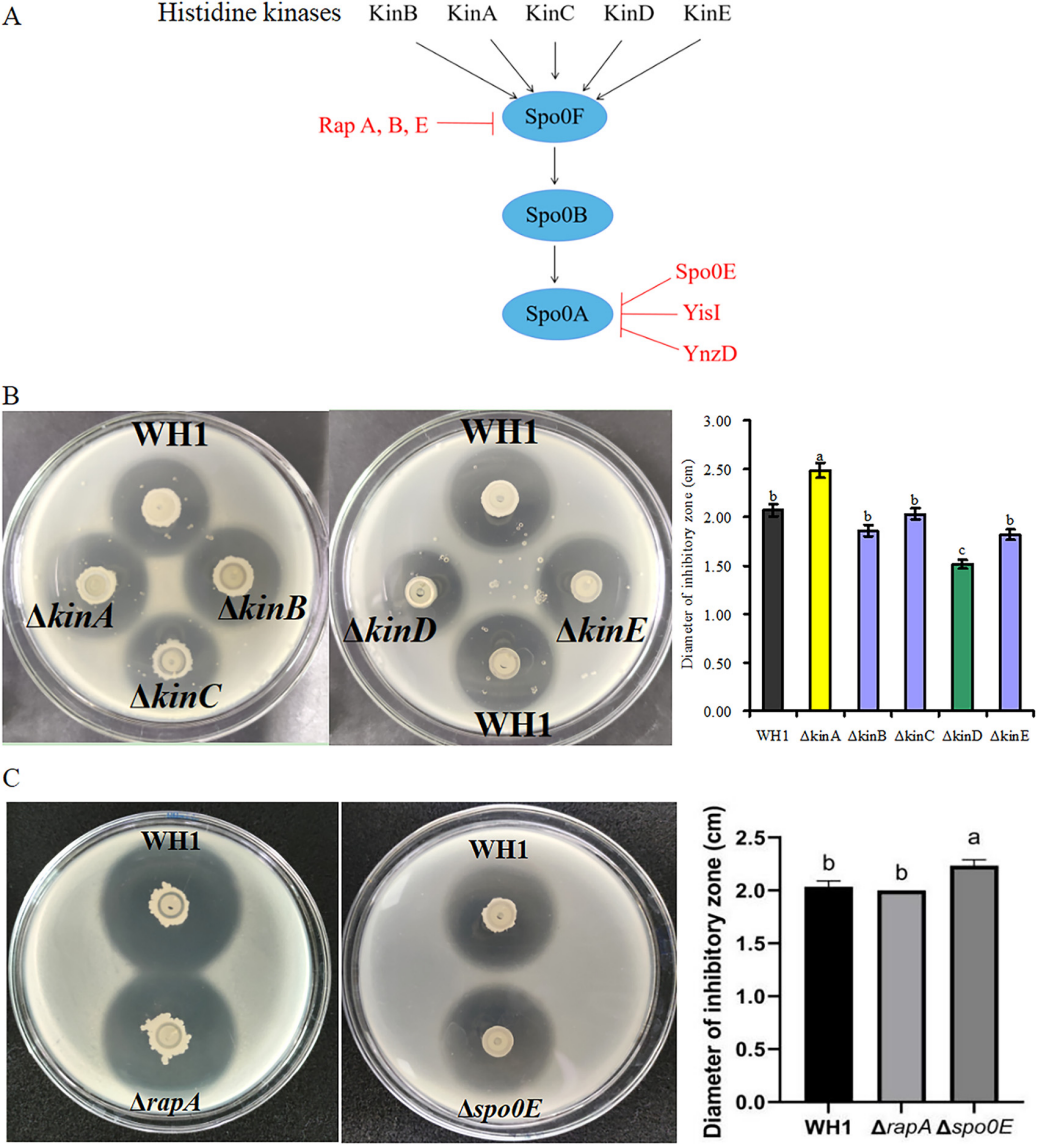
Tuning Spo0A~P level affects difficidin production. (A) Schematic diagram of phosphorylating and dephosphorylating processes of Spo0A. Histidine kinases (Kin A-E) transfer phosphate to Spo0F followed by Spo0B, and then activate Spo0A. The phosphorylated Spo0A can be dephosphorylated by RapA, Spo0E, YisI, and YnzD. (B) Comparing antibacterial activity of wild-type WH1, mutants Δ*kinA*, Δ*kinB*, Δ*kinC*, Δ*kinD*, and Δ*kinE*. (C) Comparing antibacterial activity of wild-type WH1, mutants Δ*rapA* and Δ*spo0E*. Different letters on bars indicate significant differences among groups.

Histidine protein kinases (i.e., Kin A-E) are involved in the phosphorylation of Spo0A in *Bacillus* species (13) (Fig. 6). Thereby, the mutation of histidine kinases should influence the phosphorylation levels of Spo0A, and thus affect difficidin production. The antibacterial activity of mutants Δ*kinB* (DIZ = 1.89 cm), Δ*kinC* (DIZ = 2.01 cm), and Δ*kinE* (DIZ = 1.87 cm) were all similar to that of wild-type WH1 (DIZ = 2.09 cm). The antibacterial activity of mutant Δ*kinA* increased by 19% (DIZ = 2.48 cm). The antibacterial activity of Δ*kinD* decreased by 28% (DIZ = 1.51 cm) compared to wild-type WH1. Therefore, changing the phosphorylation levels of Spo0A by deleting gene *kinA* or *kinD* affected the biosynthesis of difficidin.

Some phosphatases such as Spo0E can directly, and RapA can indirectly, dephosphorylate Spo0A~P (14). The antibacterial activity of mutant Δ*rapA* (DIZ = 1.99 cm) was similar to wild-type WH1 (DIZ = 2.01 cm). The antibacterial activity of mutant Δ*spo0E* increased by 12% (DIZ = 2.25 cm). Abolishing the role of Spo0E in dephosphorylating Spo0A~P was favorable for increasing the phosphorylation level of Spo0A, which consequently improved the difficidin production.

## DISCUSSION

*Bacillus* species produce a wide range of secondary metabolites to inhibit the growth of pathogens (22). In the present study, it was found that lipopeptides, macrolactin, bacillaene, and bacilysin were not the antibacterial substances produced by *B. amyloliquefaciens* WH1. These secondary metabolites possibly were antifungal substances or nematocidal substances. WH1 exhibited antifungal activity and nematocidal activity in our previous study (18). Therefore, *B. amyloliquefaciens* WH1 inhibited different pathogens by producing various active substances. WH1 was an efficient biocontrol agent with multiple functions being an all-rounder.

Rhizosphere microorganisms compete for ferric iron via secreted siderophore molecules, thus inhibiting phytopathogen growth (23). *B. amyloliquefaciens* WH1 could produce siderophore bacillibactin, which can seize iron from pathogens and hence inhibit the pathogen’s growth. In the environment of low iron stress, WH1 can produce siderophore bacillibactin to form iron chelates, thus indirectly inhibiting the growth of R. solanacearum by competing iron. The mechanism of how bacillibactin chelates iron in a soil environment will be studied in future research. Under conditions of iron sufficiency, ferric iron uptake transcriptional regulator Fur represses the synthesis of bacillibactin by binding the promoter of the *dhb* gene (21). Mutation of gene *fur* improved the antibacterial activity; this was because the production of bacillibactin increased in mutant Δ*fur* compared to wild-type WH1. Whether Spo0A regulates bacillibactin synthesis through repressing Fur should be investigated in future research. The siderophore bacillibactin can be used as a biocontrol agent directed against plant pathogens.

Difficidin is an effective antibacterial agent produced by *Bacillus* (24). Different *Bacillus* species produce antibacterial difficidin to inhibit plant bacterial pathogens. For instance, difficidin produced by *B. velezensis* acts efficiently against Erwinia amylovora (11). The mutation of the difficidin biosynthetic gene cluster of *B. vallismortis* resulted in a loss of antagonistic activity against R. solanacearum (4). Here, it was found that difficidin was the main antibacterial substance produced by *B. amyloliquefaciens* WH1. Whether difficidin produced by WH1 has a broad-spectrum antibacterial activity should be investigated in future research. Difficidin inhibits the protein biosynthesis of pathogenic bacteria, but the exact molecular target remains unknown (11). Cells of R. solanacearum treated with difficidin were swelled and broken, which is consistent with the previous reports (4). The exact mechanism of difficidin inhibiting R. solanacearum growth requires further study. In addition future studies should investigate whether difficidin affects the expression of genes related to virulence, protein, and cell wall synthesis of R. solanacearum.

The regulation mechanism that controls the synthesis of difficidin is unknown. In the WH1 genome, a 683 bp noncoding sequence was found upstream of the *dfn* gene cluster; therefore, it was speculated that the transcription factor should bind this region for controlling the transcription and expression of the *dfn* gene cluster. Here, it was found that Spo0A could bind to the promoter region and regulate the transcription of the *dfn* gene cluster. The exact binding site of Spo0A at promoter *P_dfn_* will be investigated in future research. It is well known that Spo0A is a global regulator that directly or indirectly regulates more than 500 genes’ transcription in *Bacillus*. Many studies have revealed that Spo0A regulates the production of secondary metabolites (15–18). The regulation role of Spo0A on difficidin synthesis was first revealed in this study. Other regulatory proteins which play a role in governing difficidin synthesis might be found in more in-depth research. Sporulation is initiated by the activation of the key transcription regulator Spo0A, and the connection between sporulation and difficidin synthesis is intriguing.

The phosphorylated protein Spo0A~P regulates the transcription of numerous genes (13). In the present study, it was found that the change in the phosphorylation level of Spo0A affected the production of difficidin. This finding was not reported in previous studies. The Spo0E phosphatase of *Bacillus* can dephosphorylate Spo0A~P (14). The phosphorylation level of Spo0A was increased in mutant Δ*spo0E*, and then the increase of Spo0A~P further improved the production of difficidin (Fig. 6). Biosynthesis of difficidin could be regulated by controlling the phosphorylation level of Spo0A. This was an important discovery for improving the antibacterial activity of WH1.

Histidine kinases (e.g., Kin A-E) activate Spo0A via the phosphate transfer proteins Spo0F and Spo0B (13). The phosphorylation level of Spo0A was possibly changed in mutants Δ*kinA* and Δ*kinD.* An increase in Spo0A~P triggers quorum-sensing responses such as sporulation and biofilm formation (13). The deletion of the *kinD* gene from the WH1 genome might decrease the phosphorylation level of Spo0A, and thus reduce difficidin production. WH1 produced secondary metabolites during the biofilm-forming period (18). Mutant Δ*kinD* produced fewer biofilm compared to wild-type WH1. It was speculated that biofilm formation was essential for producing difficidin.

In *Bacillus* species, the spore formation process is associated with the synthesis of secondary metabolites. Sensor histidine kinase KinA is involved in the phosphorelay leading to sporulation. Mutant Δ*kinA* could not form spores; therefore, it could produce more secondary metabolites such as difficidin during longer growth periods (18). As a result, the antibacterial activity of mutant Δ*kinA* significantly increased. Temporal regulation of *kinA* gene expression might increase difficidin production and subsequently form spores, which is important for the application of *Bacillus* agents. The effect of the mutation of KinA on antimicrobial activity was not reported previously. The mechanism of how histidine kinases affect difficidin biosynthesis requires further investigation.

In conclusion, difficidin and to a minor extent bacillibactin, efficiently inhibited the growth of R. solanacearum. The repressor Fur bound the promoter of the *dhb* gene cluster to suppress the biosynthesis of bacillibactin. Spo0A regulated biosynthesis of difficidin. Change in the phosphorylation level of Spo0A affected the production of difficidin.

## MATERIALS AND METHODS

### Bacterial strains and materials.

Bacterial strains were listed in Table S1. Materials for DNA manipulation were purchased from TaKaRa Bio (Beijing, China). All chemicals used were of analytical grade and supplied by Sinopharm Chemical Reagent Co., Ltd. (Beijing, China).

### Construction of mutant strains.

The genes (i.e., *srfA*, *ituA*, *fenC*, *kinA*, *kinB*, *kinC*, *kinD*, *kinE*, *baeR*, *bacA*, *dfnI*, *minH*, *dfnB*, *fur*, *dhbF*) were deleted from *B. amyloliquefaciens* WH1 genome by double-crossover homologous recombination (25). Briefly, two ~500-bp arms homologous to the 5′ and 3′ coding region of the target genes were amplified by PCR from strain WH1 and ligated by overlapping extension PCR. Then, the DNA fragments were inserted into vector T2. The resulting recombinant plasmids were electronically transformed into the competent cells of strain WH1. Knockout strains were screened out and confirmed by nucleotide sequencing. The single, double, and triple knockout strains were constructed successively. The primers for the construction of mutants were described in Table S2. The same image is being utilized to represent the colony of the wild-type WH1.

Complementary strains were constructed as follows. The corresponding gene and its promoter were amplified from the strain WH1 genome and cloned into vector T2. The recombinant plasmids were electronically transformed into knockout strains. Complementary strains were screened on LB medium plates containing 20 μg/mL kanamycin. Recombinant plasmids were transformed into wild-type WH1 to construct overexpression strains.

### Real-time reverse transcription-PCR.

The transcription levels of genes *dhbF* and *dfnI* were analyzed by real-time reverse transcription-PCR (qRT-PCR). A single colony of each strain was grown in LB medium at 37°C overnight, and then the broth was transferred into fresh LB medium at a ratio of 1% (vol/vol). After incubation at 37°C for 12 h and 36 h, the cells were collected, respectively. mRNA was isolated with RNeasy minikit (Qiagen, German). cDNA was synthesized by reverse transcription with 1 μg RNA, iScript Select cDNA Synthesis Kit and random oligonucleotide primers (Bio-Rad, USA). Using cDNA as a template, qRT-PCR was performed with SsoAdvanced Universal SYBR green Supermix (Bio-Rad, USA) and target-specific primers (Table S3) in CFX96 Real-Time PCR Detection System (Bio-Rad, USA). All expression data were normalized to the copy number of 16S rRNA in each sample (18).

### Purification and identification of difficidin.

Wild-type WH1 and mutant Δ*dfnI* were respectively cultured in 250 mL LB medium at 37°C at 180 rpm for 48 h, and then the broth was centrifuged at 8,000 g at 4°C for 10 min. The supernatant was collected and extracted with the same volume of ethyl acetate. After centrifugation at 8,000 g for 10 min, the phase of ethyl acetate was collected and dried using a rotary vacuum evaporator. The residuals were dissolved in 2 mL methanol and then separated by high-performance liquid chromatography-electrospray ionization (HPLC-ESI) as previously described (11, 26). The peak appeared in wild-type WH1 but disappeared in Δ*dfnI* broth and was identified as difficidin. The retention time of difficidin was recorded, and the eluate was collected and lyophilized to obtain difficidin. The extracted difficidin was analyzed by LC-MS.

### Detecting antibacterial activity.

R. solanacearum was cultured in LB medium at 28°C at 180 rpm overnight. The broth was mixed with LB agar medium (~42°C) at a ratio of 1: 99 (vol/vol) and poured into the petri dish. Wild-type WH1 and the engineering strains were respectively cultured in 5 mL LB medium at 37°C at 180 rpm overnight. The broths were transferred into 5 mL fresh LB medium at a ratio of 1% (vol/vol) and cultured 6 h. Then, 1 μL broth of WH1 or mutants was inoculated on the plates with R. solanacearum, and incubated at 28°C for 2 days. The diameters of the inhibition zone were measured. Experiments were conducted in triplicate.

The antibacterial activity of difficidin was determined as follows: 20 μL difficidin solution, methanol, and ethyl acetate were added to the LB agar plate inoculating R. solanacearum, respectively. After incubation at 28°C for 2 days, the diameters of the inhibition zone were measured. The effect of difficidin on the cell morphology of R. solanacearum was determined. Briefly, the broth of R. solanacearum (OD_600_ ≈ 3.0) was transferred into a 50 mL LB medium containing 45 μL purified difficidin, then cultured at 28°C at 180 rpm. After incubation for 24 h and 48 h, the broth was centrifuged at 8,000 g for 5 min. The cell precipitates were stained with sodium phosphotungstate for 30 s and then observed by transmission electron microscope (Hitachi, Japan). Cells untreated with difficidin were used as the control.

### Detecting the activity of bacillibactin.

Siderophore bacillibactin production was detected using the chrome azurol sulfonate (CAS) agar plate. Briefly, the tested strains (e.g., WH1, Δ*fur*, Δ*dhbF*, Δ*fur*Δ*dhbF*) were respectively grown in LB medium at 180 rpm at 37°C overnight. Then, 1 μL culture (2 × 10^7^ cells per mL) was spotted on CAS-agar plates and incubated at 37°C for 48 h. The orange halo formed around colonies indicating the ability of bacteria to produce siderophore (27).

### Electrophoretic mobility shift assay.

The genes *spo0A* and *fur* were respectively cloned into the expression vector pET-28a. The primers were listed in Table S4. The recombinant plasmids were transformed into E. coli BL21(DE3). The recombinant proteins were induced to express using IPTG and purified by Ni-NAT affinity chromatography (28).

Spo0A-DNA or Fur-DNA interactions and binding affinity were detected by electrophoretic mobility shift assay (EMSA). First, the fluorescent FAM-labeled DNA probes were prepared as follows. The *dfn* or *dhb* promoter region (~400 bp) was amplified by PCR. The amplified fragments were inserted into the pMD19-T vector. The recombinant plasmids pMDT-*dfn* and pMDT-*dhb* were constructed, respectively. The FAM-labeled probes were synthesized by PCR amplification using the FAM-labeled primers P*_dfn_* and P*_dhb_* (Table S5). The PCR product was purified using the Wizard SV Gel and PCR Clean-Up System (Promega, USA). Second, EMSA was performed as follows. The binding reaction system included the binding buffer (50 mM Tris-HCl, 100 mM KCl, 2.5 mM MgCl_2_, 0.2 mM DTT, 2 μg salmon sperm DNA [Sal], and 10% glycerol, pH 8.0), 50 ng probe, and the different concentrations of Spo0A or Fur proteins. Reactions were incubated at 30°C for 30 min and loaded onto a 2% agarose gel. After electrophoresis was finished, the gels were scanned using an ImageQuant LAS 4000 mini (GE Healthcare, USA) (29). Protein-nucleic acid complexes migrated more slowly in the gel than the free nucleic acid.

### Statistical analysis of data.

All experiments were repeated in triplicates. The means between the two groups were compared by the Student's *t* test. Note, * and ** represented *P < *0.05 and *P < *0.01, respectively. The means among three or more groups were compared using the analysis of variance (ANOVA) test.

### Data availability.

The genome of *B. amyloliquefaciens* WH1 was deposited in the National Center for Biotechnology Information database with accession number PRJNA906262.
