# Supplementary material for: Transcription Factor Spo0A Regulates the Biosynthesis of Difficidin in Bacillus amyloliquefaciens
Source: Microbiol Spectr. 2023 Jul 11;11(4):e01044-23. doi: 10.1128/spectrum.01044-23 (PMC10434259; doi:10.1128/spectrum.01044-23)

Table S1 Bacterial strains used in this study

Strains	Relevant properties	Source
B. amyloliquefaciens WH1	wild-type	Stored in this lab (Chen et al., 2020)
Δ*srfA*	*srfA*-knockout strain	Stored in this lab (Chen et al., 2020)
Δ*ituA*	*ituA*-knockout strain	Stored in this lab (Chen et al., 2020)
Δ*fenC*	*fenC*-knockout strain	Stored in this lab (Chen et al., 2020)
Δ*srfA*Δ*ituA*	*srfA* and *itu*-knockout strain	This study
Δ*fenC*Δ*ituA*	*fenC* and *ituA*-knockout strain	This stud
Δ*srfA*Δ*fenC*	*srfA* and *fenC*-knockout strain	This study
Δ*srfA*Δ*fenC*Δ*ituA*	*srfA, fenC* and *ituA*-knockout strain	This study
Δ*spo0A*	*spo0A*-knockout strain	Stored in this lab (Chen et al., 2020)
Δ*kinA*	*kinA*-knockout strain	Stored in this lab (Wang et al., 2022)
Δ*kinB*	*kinA*-knockout strain	Stored in this lab (Wang et al., 2022)
Δ*kinC*	*kinA*-knockout strain	Stored in this lab (Wang et al., 2022)
Δ*kinD*	*kinA*-knockout strain	Stored in this lab (Wang et al., 2022)
Δ*kinE*	*kinA*-knockout strain	Stored in this lab (Wang et al., 2022)
Δ*baeR*	*baeR*-knockout strain	This study
Δ*bacA*	*bacA*-knockout strain	This study
Δ*dfnI*	*dfnI*-knockout strain	This study
Δ*dhbF*	*dhbF*-knockout strain	Stored in this lab (Wang et al., 2022)
Δ*minH*	*minH*-knockout strain	This study
Δ*sfp*	*sfp*-knockout strain	Stored in this lab (Wang et al., 2022)
Δ*dfnB*	*dfnB*-knockout strain	This study
Δ*fur*	*fur*-knockout strain	Stored in this lab (Wang et al., 2022)
Δ*fur*Δ*dfnI*	*fur* and *dfnI*-knockout strain	This study
Δ*fur*Δ*dhbF*	*fur* and *kinA*-knockout strain	This study
Δ*spo0A*/T2-*spo0A*	Complementary strain with *spo0A*	Stored in this lab (Wang et al., 2022)
WH1/T2-*spo0A*	Overexpression of *spo0A*	Stored in this lab (Wang et al., 2022)

Table S2 Primers for construction of knockout strains

Name	Primer sequence (5'-3')	Purpose
srfA-LF	CG**GGATCC**GAATCTTTTTGAAGCGCTCT	Amplifying L arm of *srfA* and verification of double-crossover
srfA-LR	ATATAAAGCATGTGTGCGCCTCCCCTTTT	
srfA-RF	CGCACACATGCTTTATATCGTGCCGAAAA	Amplifying R arm of *srfA* and verification of double-crossover
srfA-RR	GC**TCTAGA**CGGACTTTCACCTGATCATC	
srfA-dan-LF	TTATGCCGATTTTGGACGCCAT	Amplifying L arm of *srfA* and verification of single-crossover
srfA-dan-RR	TGCCCTGCTGATCCGGCCGT	
fenA-LF	CG**GGATCC**CTTCGGAGCCATTTGATATA	Amplifying L arm of *fenA* and verification of double-crossover
fenA-LR	CTTGCTGGCTTTCATAAAAAGGTGTGTGG	
fenA-RF	TTTATGAAAGCCAGCAAGCCTGATCTCCG	Amplifying R arm of *fenA* and verification of double-crossover
fenA-RR	GC**TCTAGA**TGGCGAGTTTCTCATTTGA	

fenA-dan- LF	GTTTTGCGCCGTCTTATTCT	Amplifying L arm of *fenA* and
fenA-dan- RR	AGGGAGTCGAAGTCAGAAAT	verification of single-crossover
ituB-LF	CG**GGATCC**TGCTCTAGTGAAACAACTGT	Amplifying L arm of *ituB* and
ituB-LR	TTGTTCCGCTCACGGGGGCAGCGGCTGTC	verification of double-crossover
ituB-RF	GCCCCCGTGAGCGGAACAAAAGTTTCGAGT	Amplifying R arm of *ituB* and
ituB-RR	GC**TCTAGA**TGACGAATCTGCTCGCTTAT	verification of double-crossover
ituB-dan-LF	AGCATCTGCAGCAGCCGTT	Amplifying L arm of *ituB* and
ituB-dan-RR	CAGCTGGAGCACAAGACGAT	verification of single-crossover
kinA-LF	CG**GGATCC**AAGTACACGTATAACC	Amplifying L arm of *kinA* and
kinA-LR	GGCATAACGCCGTAACTTTCAACC	verification of double-crossover
kinA-RF	GGTTGAAAGTTACGGCGTTATGCC	Amplifying R arm of *kinA* and
kinA-RR	GC**TCTAGA**TTTGTCCTGTCTGATGAGAT	verification of double-crossover
kinA-dan-LF	TATTCAACATTCCAAGGTTCCGGC	Amplifying L arm of *kinA* and
kinA-dan-RR	TCGCTGACACCAAAAACAAAATGC	verification of single-crossover
kinB-LF	CG**GGATCC**CATTGCATCAGAAC	Amplifying L arm of *kinB* and
kinB-LR	GACTGAAGCCGTGAAGACTTGATA	verification of double-crossover
kinB-RF	TATCAAGTCTTCACGGCTTCAGTC	Amplifying R arm of *kinB* and
kinB-RR	GC**TCTAGA**TTCAAGCTTGTCGA	verification of double-crossover
kinB-dan-LF	GTTTGCGCGGATTTCCATTACTTG	Amplifying L arm of *kinB* and
kinB-dan-RR	ATATAAAAACTTCTTTGGCGACAG	verification of single-crossover
kinC-LF	CG**GGATCC**GTTTCCGGCGGGAATGAAAT	Amplifying L arm of *kinC* and
kinC-LR	AAGAAGCAAGAGTCCCACCTGCCGC	verification of double-crossover
kinC-RF	AGGTGGGACTCTTGCTTCTTTTTGT	Amplifying R arm of *kinC* and
kinC-RR	GC**TCTAGA**CGCGGGACGGTATGTATGGT	verification of double-crossover
kinC-dan-LF	TTCCGATCTCAGTTAATTGA	Amplifying L arm of *kinC* and
kinC-dan-RR	ACGGCAAACTCTTTTCTCAT	verification of single-crossover
kinD-LF	CG**GGATCC**AATAACCCAATCAAACTT	Amplifying L arm of *kinD* and
kinD-LR	TTAAAGGTGCCCCAAAGCGAAGTC	verification of double-crossover
kinD-RF	GACTTCGCTTTGGGGCACCTTTAA	Amplifying R arm of *kinD* and
kinD-RR	GC**TCTAGA**TCACTCAGCACACT	verification of double-crossover
kinD-dan-LF	TGCCGCATTCTGTTTATATC	Amplifying L arm of *kinD* and
kinD-dan-RR	TGATACAACCTAGAAACGCC	verification of single-crossover
kinE-LF	CG**GGATCC**GAAAAAGACTTTAACT	Amplifying L arm of *kinE* and
kinE-LR	AGAAATCAAAATGCACGATGCCGT	verification of double-crossover
kinE-RF	ACGGCATCGTGCATTTTGATTTCT	Amplifying R arm of *kinE* and
kinE-RR	GC**TCTAGA**GGACAAAAATCGGCAG	verification of double-crossover
kinE-dan-LF	CATCCACTGCATGATTATTTCCTG	Amplifying L arm of *kinE* and
kinE-dan-RR	GGATATTTGTTCGATGTTCAGCAG	verification of single-crossover

baeR-LF	CG**GGATCC**GAAAACATAACAGCGC	Amplifying L arm of *baeR* and verification of double-crossover
baeR-LR	GACCCCGTAAATGTTTTTCCTCCG	
baeR-RF	CGGAGGAAAAACATTTACGGGGTC	Amplifying R arm of *baeR* and verification of double-crossover
baeR-RR	GC**TCTAGA**GCCTCGGCATCCTTCAG	
baeR-dan-LF	CGCAACAATCACTGGTACAAAC	Amplifying L arm of *baeR* and verification of single-crossover
baeR-dan-RR	ACTCCATTTTGTTTTCGCTGAC	
bacA-LF	CG**GGATCC**TATTTCTTCAGGCTTG	Amplifying L arm of *bacA* and verification of double-crossover
bacA-LR	CGCATACAGAAGAAAACCGTCCTC	
bacA-RF	GAGGACGGTTTTCTTCTGTATGCG	Amplifying R arm of *bacA* and verification of double-crossover
bacA-RR	GC**TCTAGA**GCCGTGTCCCTGTATATC	
bacA-dan-LF	CCAAGGTCAGCGATAACCAATAC	Amplifying L arm of *bacA* and verification of single-crossover
bacA-dan-RR	GGTCGCTCGCTTTGATTTTC	
dfnI-LF	CG**GGATCC**GGTGCGTTTTCATTTT	Amplifying L arm of *dfnI* and verification of double-crossover
dfnI-LR	GGCCTTGTCGCGTAACAGATTGGA	
dfnI-RF	TCCAATCTGTTACGCGACAAGGCC	Amplifying R arm of *dfnI* and verification of double-crossover
dfnI-RR	GC**TCTAGA**CGCTCTCTTTCATCCC	
dfnI-dan-LF	CATCGACTCCCTGCACCCACATTT	Amplifying L arm of *dfnI* and verification of single-crossover
dfnI-dan-RR	TGTTTCCTTGATATTCGGCAGTCT	
mlnH-LF	CG**GGATCC**TTTCAAAAGAATATAC	Amplifying L arm of *mlnH* and verification of double-crossover
mlnH-LR	GATACACAACAGCTCTTCCTTCCG	
mlnH-RF	GGATTCTAAACAGCATTATTGGAG	Amplifying R arm of *mlnH* and verification of double-crossover
mlnH-RR	GC**TCTAGA**GGGTCTTCTTCGTATT	
mlnH-dan-LF	CGGCCTGATTCATAAAGATT	Amplifying L arm of *mlnH* and verification of single-crossover
mlnH-dan-RR	AAGCCGATCGTAGAATAGCC	
dfnB-LF	CG**GGATCC**GAGAAAGCAGAAAACG	Amplifying L arm of *dfnB* and verification of double-crossover
dfnB-LR	CAAAAATACATGGATAACGGAAAAG	
dfnB-RF	CTTTTCCGTTATCCATGTATTTTTG	Amplifying R arm of *dfnB* and verification of double-crossover
dfnB-RR	GC**TCTAGA**GCCATATCGGGTTCCTG	
dfnB-dan-LF	GTTCGGATATTCAAACAGCA	Amplifying L arm of *dfnB* and verification of single-crossover
dfnB-dan-RR	GACGTCACACAAACAGAAGG	
fur-LF	CG**GGATCC**GTCCTTTATATGCTTT	Amplifying L arm of *fur* and verification of double-crossover
fur-LR	GAGTCATGGAATGGTTTTTTGCTG	
fur-RF	CAGCAAAAAACCATTCCATGACTC	Amplifying R arm of *fur* and verification of double-crossover
fur-RR	GC**TCTAGA**AAATGTTTTGGCAGGC	
fur-dan-LF	CACAATCCGCTCTTTCCGCC	Amplifying L arm of *fur* and

fur-dan-RR	CGGGGGTTCTTTTTTTATGC	verification of single-crossover
T2-F	TTAACGAATTCCTGCAGCCC	Verification of positive transformants
T2-R	TTTTCTACGAGCTCCTCCGC	
T2-dan-LR	TTGTTTGCAAGCAGCAGATT	Verification of single-crossover
T2-dan-RF	CGAAAAACAAGTTAAGGGAT	

The bold letters represent the *BamH* I and *Xba* I cleavage site sequence, respectively.

Table S3 Primers used in qRT-PCR

Name	Primer sequence (5'-3')	Purpose
16s-qF	CTGCCTGTAAGACTGGGATAAC	Amplifying 16 S rRNA sequence
16s-qR	CATCTGTAAGTGGTAGCCGAAG	
dhbF-qF	CCGTCCCATCATCGGTAAAC	Amplifying *dhbF* gene
dhbF-qR	AGCACGGCTTATCTTTCTGATC	
dfnI-qF	TATGATTTTTTTACGGATGC	Amplifying *dfnI* gene
dfnI-qR	AGTGCCGAACGCTTTACCGC	

DNA amplified here was a partial fragment, ~ 100 bp.

Table S4 Primers for construction of recombinant protein expression vectors in *E. coli*

Name	Primer sequence (5'-3')	Purpose
T7-F	TAATACGACTCACTATAGGG	Selecting positive transformants
T7-R	TGCTAGTTATTGCTCAGCGG	
fur-F	GGATCCATGGAAAACCGTATTGATCG	Amplifying *fur* gene
fur-R	GAATTCTTACTCTGTTTCATCCCC	
spo0A-F	GGATCCGTGGAGAAAATTAAAGTTTG	Amplifying *spo0A* gene
spo0A-R	GTCGACTTACGAAGCTTTATGCTC	

Underlined letters represent the *BamH* I (GGATCC), *Eco*R I (GAATTC), and *Sal* I (GTCGAC) cleavage site sequence, respectively.

Table S5 Primers for amplifying EMSA Probes

Primer	Sequence (5'-3')	Purpose
P_{dfn}	AAAATAACGAAATAAAGCCT	EMSA for *dfn*
P_{dhb}	GAATCATAATTGATAATGATAATCATTATC	EMSA for *dhb*

Fig. S1 Expression and purification of recombinant proteins. A: Recombinant Fur protein; **B:** Recombinant Spo0A protein. Lane M: Protein markers; lane 1: Purified recombinant protein. Arrows direct the purified recombinant proteins, respectively.

A B

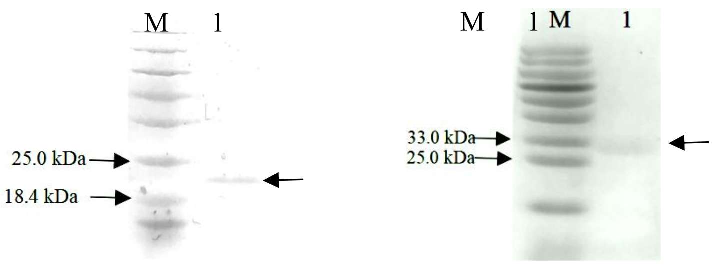

Supplement: Supplemental file 1 — Tables S1 to S5 and Fig. S1. Download spectrum.01044-23-s0001.pdf, PDF file, 0.2 MB [file spectrum.01044-23-s0001.pdf]
